# Ventromedial prefrontal value signals and functional connectivity during decision-making in suicidal behavior and impulsivity

**DOI:** 10.1038/s41386-020-0632-0

**Published:** 2020-02-08

**Authors:** Vanessa M. Brown, Jonathan Wilson, Michael N. Hallquist, Katalin Szanto, Alexandre Y. Dombrovski

**Affiliations:** 1 Department of Psychiatry, University of Pittsburgh, Pittsburgh, PA USA; 2 Department of Psychology, Pennsylvania State University, State College, PA USA

**Keywords:** Learning and memory, Depression

## Abstract

Suicide is linked to impaired value-based decision-making and impulsivity, but whether these risk factors share neural underpinnings is unclear. Disrupted ventromedial prefrontal cortex (vmPFC) value signals may underlie this behavioral phenotype. We investigated vmPFC value signals, vmPFC–frontoparietal connectivity, and the impact of impulsivity during decision-making in depressed individuals with and without suicidal behavior. Middle-aged and older adults (*n* = 116; 35 with a history of suicide attempts, 25 with ideation only, 25 depressed controls with no ideation, and 31 nonpsychiatric controls) completed a decision-making task with drifting reward probabilities during fMRI. Values of choices, estimated by a reinforcement learning model, were regressed against BOLD signal. VmPFC value activation was compared between groups. Moderating effects of impulsivity on vmPFC–frontoparietal connectivity were assessed in nonpsychiatric controls and compared among patient groups. VmPFC value responses in participants with a history of suicide attempts were reduced relative to nonpsychiatric controls (*p* < 0.05). In nonpsychiatric controls, vmPFC–frontoparietal connectivity was negatively moderated by impulsivity (*p*_FWE corrected_ < 0.05). This effect was preserved in comparison patient groups but abolished in suicide attempters (*p* < 0.001). This change in neural connectivity patterns also affected behavior: people with a history of suicide attempts showed a disrupted effect of vmPFC–frontoparietal connectivity, impulsivity, and reinforcement on choice quality (*p* < 0.001). These effects were specific to vmPFC and not to striatum. In summary, findings from this study largely support disrupted vmPFC value signals in suicidal behavior. In addition, it uncovers an altered pattern of vmPFC–frontoparietal connectivity in impulsive people with suicidal behavior, which may underlie disrupted choice processes in a suicidal crisis.

## Introduction

An accumulating body of evidence suggests that suicidal behavior is facilitated by impaired decision-making, particularly in complex and dynamically evolving critical situations [1]. Both impaired value-based decision-making in the laboratory [2] and self-reported impulsivity [3] are associated with suicidal behavior. On a neural level, we have found disrupted ventromedial prefrontal cortex (vmPFC) value encoding in people who have attempted suicide [4]. This disrupted value encoding scaled with trait impulsivity, suggesting that decision-making deficits and impulsivity may represent overlapping manifestations of a single neural diathesis to suicidal behavior.

Computational models based on formal learning theory can uncover precise disruptions in decision processes while ruling out alternate explanations for decision deficits [5]. In suicidal behavior, disrupted components of decision-making include difficulty choosing between similarly valued options and impaired encoding of recent reinforcement [6, 7], revealed by computational modeling of decision task data. Notably, decision-making alterations appear to be selective to people who have engaged in suicidal behavior, not generally to suicidal ideation or depression [2, 8].

Disrupted neural processing of value may also relate to elevated impulsivity in people who attempt suicide. Impulsivity is linked to suicidal behavior, but self-reported impulsivity alone does not distinguish ideation from attempt [9, 10]. Therefore, understanding how disruptions in neural processing of value connect to impulsivity and, in turn, suicidal behavior will improve understanding of risk factors for suicidal behavior. Deficits in value-based decision-making found in suicide suggest that facets of impulsivity related to escaping shorter-term negative situations vs. persisting with actions higher in long-term value (i.e., negative urgency or the tendency to act rashly during negative mood states; [11]) may best map on to neural correlates of disrupted value in suicide. In the general population, more impulsive individuals exhibit weaker connectivity among frontoparietal regions, particularly dorsolateral and ventrolateral PFC [12–14], consistent with reduced frontoparietal cognitive control. During decision-making, failure to engage cognitive control, for example, in highly impulsive individuals, is linked to reduced connectivity of these frontoparietal cognitive control regions with brain areas representing expected reward value, particularly vmPFC [15, 16]. Therefore, one putative neural correlate of impulsive decision-making is reduced connectivity of frontoparietal regions with vmPFC and other areas encoding value. Reduced vmPFC–frontostriatal connectivity may result in reduced cognitive control and selection of choices based on proximal vs. distant outcomes, similar to behavior during a suicidal crisis. However, whether this impulsivity-related pattern of connectivity is characteristic of suicidal behavior has yet to be investigated. In addition, impulsivity is linked to disrupted striatal functioning and connectivity [17–21], but this neural pathway has been less studied in suicide.

In summary, two candidate neural mechanisms may underlie impaired decision-making and impulsivity in suicidal behavior, which are not mutually exclusive. The first is the disruption of value representations in the vmPFC observed in impulsive suicide attempters [4]. The second, altered functional connectivity between the vmPFC and frontoparietal cognitive control regions [15, 16], relates to impulsivity independent of suicide. Thus altered vmPFC value encoding and frontoparietal connectivity may define a single underlying vulnerability, which manifests pleiotropically as suicidal behavior and impulsivity. Alternatively, the neural diathesis toward impulsive suicidal behavior may be distinct from the general neural correlates of impulsivity. Understanding whether these neural mechanisms are shared vs. distinct, and how they relate to other neural mechanisms implicated in impulsivity such as striatal connectivity, will inform the biological classification of suicidal behavior and neuroscience-based interventions.

To arbitrate between these possibilities, we need to examine decision processes like those that unfold during a suicidal crisis in impulsive and non-impulsive people who have attempted suicide. Although earlier work links neural value computations to suicidal behavior and suggests that they are modulated by impulsivity (e.g., [4]), no previous study has (1) fully isolated disrupted value signals from other learning deficits, (2) investigated correlates of suicidal behavior above and beyond ideation, and (3) measured impulsivity along facets (i.e., negative urgency) that distinguish attempters from ideators [10, 22] and that reflect the impact of intense negative emotions, as during a suicidal crisis. The current study sought to fill these gaps. Furthermore, we focused on suicide attempts in older adults, as they are more representative of death by suicide [23] and occur in the context of a wider range of impulsivity [24]. Disrupted decision processes may play a particularly prominent role in the emergence of suicidal behavior in older adults, in whom latent cognitive vulnerabilities undermine decision competence [25]. Finally, whereas younger people who attempt suicide are predominantly impulsive, older suicide attempters show a broader range from very low to very high impulsivity [24]. To examine decision processes, we assessed neural encoding of value while participants chose between multiple options that dynamically and independently varied in reward probabilities. We examined the neural signatures of suicidal behavior and trait impulsivity, specifically negative urgency, in two regions central to value-based decision-making, vmPFC and striatum.

## Materials and methods

### Participants

Participants were middle-aged and older adults (ages 47–79 years) recruited for a longitudinal study of suicidal behavior in late-life depression [26] from a psychogeriatric inpatient unit, late-life depression clinic, primary care clinics, and community advertisements in the Pittsburgh, PA region. See Supplementary Materials for further details on inclusion/exclusion criteria and verification of attempter status. All participants provided informed consent and study procedures were approved by the University of Pittsburgh Institutional Review Board. Table 1 contains details on participant characteristics.

**Table 1:** Participant characteristics.

	Nonpsychiatric controls	Depressed controls	Ideators	Attempters	Omnibus *F* or *χ*^2^ test	*p*
*N*	31	25	25	35	—	—
Sex (# [%], female)	19 (61.3)	13 (52.0)	11 (44.0)	20 (57.1)	*χ*^2^_3_ = 1.85	0.6
Age	62.0 (9.5)	61.9 (8.0)	60.1 (5.7)	61.2 (7.1)	*F*_3,112_ = 0.34	0.8
Race (# [%])					*χ*^2^_9_ = 10.5	0.31
African American	2 (6.4)	6 (24.0)	2 (8.0)	6 (17.1)		
Asian	0 (0)	1 (4.0)	0 (0)	1 (2.9)		
Caucasian	29 (93.5)	18 (72.0)	22 (88.0)	28 (80.0)		
Multiracial	0 (0)	0 (0)	1 (4.0)	0 (0)		
Education (years)	16.4 (2.6)	15.1 (2.4)	15.5 (1.8)	15.1 (2.9)	*F*_3,112_ = 1.82	0.15
Highest attempt lethality	—	—	—	3.0 (2.0)	—	—
Impulsivity (UPPS)						
Negative urgency	17.6 (4.2)^a^	24.8 (6.7)^b^	29.5 (8.3)^b^	29.4 (7.9)^b^	*F*_3,106_ = 19.3	<0.001
Positive urgency	16.4 (3.7)^a^	21.6 (7.6)^b^	23.8 (7.5)^b^	24.5 (8.0)^b^	*F*_3,106_ = 8.5	<0.001
Lack of premeditation	19.8 (4.3)	21.5 (5.5)	23.4 (6.9)	22.9 (7.0)	*F*_3,106_ = 2.05	0.11
Lack of perseveration	15.4 (2.5)^a^	22.6 (4.5)^b^	22.5 (5.5)	20.3 (6.0)	*F*_3,106_ = 13.2	<0.001
Cognitive control (EXIT)	4.6 (2.7)	5.5 (3.3)	5.6 (2.9)	6.3 (2.8)	*F*_3,106_ = 1.7	0.17
Dementia Rating Scale	139.0 (2.7)^a^	136.3 (5.7)^a,b^	136.5 (4.0)^a,b^	135.9 (4.0)^b^	*F*_3,104_ = 3.21	0.026
Estimated IQ (WTAR)	108.4 (8.9)	107.1 (15.1)	110.8 (15.3)	105.1 (15.1)	*F*_3,103_ = 0.85	0.47
Lifetime substance abuse (# [%])	—	10 (40.0)	14 (56.0)	20 (57.1)	*χ*^2^_2_ = 1.97	0.37
Current substance abuse (# [%])	—	2 (8.0)	3 (12.0)	7 (20.0)	*χ*^2^_2_ = 1.86	0.39
Anxiety disorder (# [%])	—	9 (36.0)	14 (56.0)	26 (72.3)	*χ*^2^_2_ = 8.80	0.012
Potential brain damage (# [%])	—	—	—	3 (8.6)	—	—
Medication use: (# [%])						
Antipsychotics	0 (0)	1 (4.0)	4 (11.4)	9 (36.0)	*χ*^2^_3_ = 12.3	0.009
Sedatives	0 (0)	3 (12.0)	2 (8.0)	3 (8.6)	*χ*^2^_3_ = 3.51	0.39
Opiates	1 (3.2)	3 (12.0)	2 (8.0)	7 (20.0)	*χ*^2^_3_ = 4.98	0.2
HRSD-16 at baseline	2.0 (2.3)^a^	17.8 (4.0)^b^	19.1 (6.2)^b,c^	21.3 (6.0)^c^	*F*_3,112_ = 100.5	<0.001
HRSD-16 at scan	2.2 (2.1)^a^	13.2 (5.3)^b^	14.1 (6.4)^b^	14.7 (8.3)^b^	*F*_3,112_ = 29.3	<0.001
Suicidal intent	0 (0)^a^	0.3 (1.2)^a^	15.7 (6.8)^b^	21.2 (9.0)^c^	*F*_3,105_ = 99.5	<0.001

For continuous measures with significant group differences, groups sharing the same superscripted letter do not significantly differ (*p* < 0.05, Tukey’s HSD correction).

*EXIT* Executive Interview, *WTAR* Wechsler Test of Adult Reading, *HRSD-16* Hamilton Rating Scale for Depression, 17-item version without suicide item, *Suicidal intent* Beck Scale for Suicidal Ideation.

Participants were recruited into four groups: suicide attempters, suicide ideators, depressed non-suicidal controls, and non-depressed non-suicidal controls. Suicide attempters were required to have depression with a history of self-injurious act(s) with the intent to die within the month prior to study enrollment or a history of suicide attempt(s) with strong current suicidal ideation at the time of study enrollment. Suicide ideators with depression were included to identify specific correlates of suicidal behavior above and beyond ideation. This group allowed us to test the relationship between impulsivity and value-based decision-making in people who have attempted suicide vs. those who have suicidal ideation without making an attempt. This comparison is crucial for understanding the role trait impulsivity and dynamic decision processes play in the transition from ideating about suicide to engaging in suicidal behavior [27]. Participants in this group had suicidal ideation but no history of attempt. To be included in this group, participants were required to have active ideation with a specific plan; people with a passive death wish or ambiguous or transient ideation were excluded. Non-suicidal depressed individuals were included to identify correlations of suicidal ideation and behavior above and beyond depression and had no lifetime history of self-injurious behavior, suicidal ideation, or suicide attempts, as assessed by self-report during clinical assessment, review of medical records, responses on the Structured Clinical Interview for DSM-IV (Diagnostic and Statistical Manual of Mental Disorders, fourth edition) (SCID), and a score of 0 on the 17-item Hamilton Rating Scale for Depression (HRSD-17) suicide item. Nonpsychiatric controls had no lifetime history of psychiatric disorders as assessed by the SCID. Except for nonpsychiatric controls, all participants were required to score ≥14 on the HRSD-17 at study entry.

### Procedures

#### Measures

Diagnosis of depression and other psychiatric disorders was ascertained with the SCID [28]. Severity of depression was assessed with the HRSD-17 version [29]. Impulsivity was assessed with the UPPS [11], which consists of four scales measuring negative urgency, positive urgency, lack of premeditation, and lack of perseveration. See Supplementary Materials for additional measures used in sensitivity analyses.

#### Reinforcement learning task

Participants completed 300 trials of a three-armed bandit task [30] during functional magnetic resonance imaging (fMRI) scanning (median days between baseline assessment and scanning = 84.5). This task used outcomes of drifting reward probabilities to enable continuous learning and updating of value throughout the task, requiring participants to incorporate long-term value estimates (from gradual reinforcement learning) with short-term value (from immediate reinforcement). Therefore, it provides a more sensitive and cognitively demanding assessment of value-based learning than tasks previously used to study this process in suicide (e.g., [4]), which are primarily serial reversal paradigms that may lead to pattern matching or other forms of non-value-based learning. Participants chose among three abstract stimuli that varied in their probability of reinforcement. Participants who did not engage in the task (*n* = 2: one depressed control and one ideator; defined as making the same button press >10 times in a row) were excluded from analyses.

See Supplementary Material for fMRI data collection, preprocessing, and first-level analysis information.

### Data analysis

#### Behavioral data analysis

Behavioral choices were fit to a reinforcement learning model (see Supplementary Materials and [6] for details on behavioral model fitting and model comparison). To investigate choice behavior related to altered neural processing, the best possible outcome (maximum available value) at each trial was calculated from the best fitting parameters for each subject. Full behavioral results from this sample are reported in [6]; briefly, participants with a history of suicidal behavior were less responsive to reinforcement and struggled to distinguish options close in value.

#### fMRI generalized linear model analysis

To measure neural responses to expected value in vmPFC, the beta value of the expected value regressor was extracted from a meta-analytically defined region of interest (ROI) [31] representing areas active to reward value. A meta-analytically defined ROI was used over a mask created from control participants’ value signals because of the consistency of vmPFC responses to value across tasks and to increase power for this analysis. These beta values were compared between people with a history of suicide attempts and the other three groups (ideators, depressed controls, and nonpsychiatric controls). Comparison analyses assessed value responses in right and left striatal seeds from the same meta-analysis. All whole-brain analyses used nonparametric thresholding to control false positive rates (*p* < 0.05 corrected with a cluster forming threshold of *p* < 0.001 using command 3dttest ++ with –Clustsim option; AFNI version AFNI_18.0.09, compiled 1/19/2018).

#### fMRI psychophysiological interaction (PPI) analysis

To test connectivity between vmPFC and other brain regions during value signals, a PPI was constructed using a generalized PPI approach [32]. This PPI assessed which voxels showed a higher correlation with the vmPFC at the feedback time point relative to other time points. The interaction of the deconvolved time course of vmPFC activity, defined using the meta-analytically derived value ROI used above, and the feedback time point was regressed against fMRI blood-oxygen-level-dependent signal along with main effects of vmPFC time course and feedback time point. To test the relationship between impulsivity and this vmPFC value connectivity, UPPS Negative Urgency scores were added as a subject-level regressor. Therefore, this interaction shows the brain regions for which the correlation between the vmPFC seed at feedback (relative to other time points) was modulated by impulsivity. To investigate impulsivity-modulated connectivity in the patient groups, an independent functional mask was created from brain areas whose connectivity was modulated by impulsivity in the nonpsychiatric control group (thresholded at *p* < 0.001 uncorrected; note that this mask was created in an independent group from the patient groups it was subsequently tested in). UPPS Negative Urgency scores were then regressed against the beta value in this independent ROI in the remaining three groups (attempters, ideators, and depressed controls), with a Group × Negative Urgency interaction testing whether modulation of vmPFC value connectivity to these regions by impulsivity differed by group, with follow-up analyses comparing the attempter and depressed control groups. To determine whether all regions in this control group-derived ROI mask showed a similar pattern of activations in participants in the patient groups, a principal components analysis was run on the beta values for each participant from the 13 clusters that comprised this mask. A one-component solution (explaining 69% of the total variance) fit best, confirming that all regions were best assessed in a single mask. Therefore, subsequent analyses used the average beta value from all clusters in the mask. To test whether striatal value signals showed similar patterns, PPI analyses were additionally carried out with right and left striatum, instead of vmPFC, as seeds.

#### PPI–behavior interaction analysis

To investigate the behavioral effects of vmPFC value connectivity on the quality of choices, participants’ beta values from a mask, independently defined by impulsivity-related connectivity in the nonpsychiatric controls, were extracted. These average beta values were entered in a hierarchical linear regression using the R *lme4* package [33]. The value of participants’ actual choices on each trial, as estimated by the reinforcement learning model, was used to analyze the effect of vmPFC value connectivity on behavior. This choice value was predicted by the interaction of beta values of the connectivity between vmPFC and the functionally defined mask, group status, and the presence of reinforcement on the immediately preceding trial. Effects of group status were tested by comparing models with a three-way interaction of group status, previous reinforcement, and connectivity to those without an interaction of previous reinforcement and connectivity with group. If the model with the three-way interaction provided a significantly better fit, follow-up analyses focused on differences between the depressed control and attempter groups. To avoid circularity, these analyses were run in the three patient groups only (attempters, ideators, and depressed controls), excluding the nonpsychiatric controls who were used to generate the functional mask.

## Results

### vmPFC value signals

**Fig. 1:**
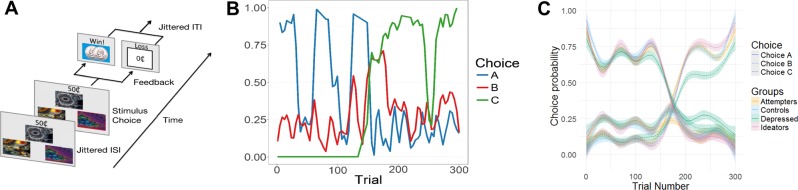
Volatile reinforcement learning task and participants’ performance. **a** Task schematic. On each trial in the task, participants were presented with three stimuli and the possible winnings (10, 25, or 50¢) if the chosen stimulus was rewarded. Reward magnitude was manipulated independently of the chosen stimulus and shown at trial onset. Stimuli and possible winnings were presented until participants made a response using MRI-compatible response gloves. After an option was selected, it was highlighted and the presence or absence of a reward was displayed after a jittered ISI (sampled from an exponential distribution with mean = 4000 ms); reward feedback was displayed for 750 ms. The intertrial interval was sampled from an exponential distribution with a mean of 2920 ms. **b** Reward probabilities by trial and stimulus. The probability of reward after choosing each stimulus varied dynamically over time, requiring participants to continually update the expected values. Colored lines indicate the probability of reward (*y* axis; 0–1) for each stimulus at each trial (*x* axis; 1–300). **c** Task performance by groups. All participants were able to track the changing probabilities and choose stimuli that reflected updated reward probabilities. Line types represent choices (A–C, corresponding to lines in **b**) and line colors represent participant groups.

**Fig. 2:**
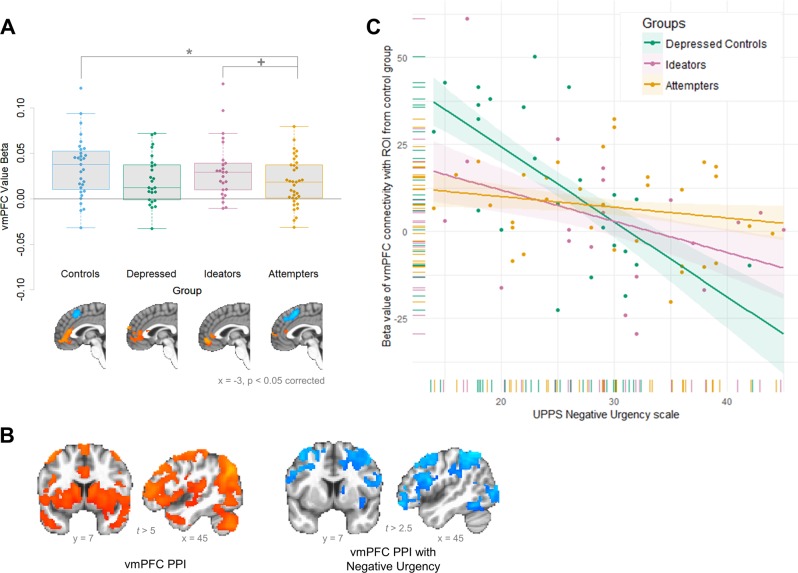
Neural processing of value in vmPFC by group and moderation of connectivity by impulsivity. **a** Ventromedial PFC value signals by group. Box plots are grouped by patient group and individual dots represent each participant in the group. Values shown are the average beta value for expected value in a meta-analytically defined vmPFC region of interest. Participants with a history of suicide attempts showed reduced vmPFC activation to value relative to nonpsychiatric controls and moderately reduced relative to participants with suicidal ideation but no history of attempts. **b** Ventromedial PFC connectivity with value and moderated by impulsivity in nonpsychiatric controls. In controls, vmPFC signal at the time of feedback was significantly correlated with activity in cortical and subcortical brain areas (left); frontoparietal regions were significantly negatively correlated with greater negative urgency scores in this group (right). **c** Moderation of relationship between vmPFC–frontoparietal connectivity with impulsivity by group. *X* axis represents impulsivity scores (UPPS negative urgency) and *Y* axis represents connectivity between vmPFC and region of interest showing altered connectivity with impulsivity in nonpsychiatric controls (mask derived from regions shown in **b**, left). Dots indicate individual participants and lines indicate overall relationship per group; colors indicate patient groups. Connectivity in participants with a history of suicide attempts (yellow) is not affected by impulsivity, in contrast to other patient groups.

Participants completed a three-armed bandit task requiring continual learning and updating of expected value (Fig. 1; Fig. S1 shows performance by diagnostic category) while undergoing fMRI scanning. When examining neural signals during learning, participants with a history of suicide attempts showed moderately reduced activation to expected value in a meta-analytically defined vmPFC ROI (Fig. 2a; *t* test vs. nonpsychiatric controls: *t*_64_ = 2.24, *p* < 0.05; vs. ideators: *t*_58_ = 1.76, *p* < 0.1; vs. depressed controls: *t*_58_ = 0.35, *p* > 0.1; *F*-test over all groups: *F*_3,112_ = 2.22, *p* = 0.08). Among participants with a history of suicide attempts, the strength of this neural value signal did not differ by the lethality of attempts, age of onset of suicidal behavior, or level of impulsivity (all *p*s > 0.1) and were similar when excluding people with brain damage, current or lifetime substance use, and treatment with antipsychotic medication. These results suggest that participants with a history of suicide attempts were selectively impaired in updating and processing learned value in the vmPFC relative to nonpsychiatric controls; however, this analysis does not assess how this vmPFC value signal relates to processing in other regions involved in decision-making.

### vmPFC functional connectivity

To assess how vmPFC neural signals modulated other brain regions, a PPI assessed the relationship between activation in this area and the rest of the brain at the time when value estimates were updated. In nonpsychiatric controls, the vmPFC ROI showed strong connectivity with limbic and frontoparietal networks during value updating (Fig. 2b left; corrected *p* < 0.05; Table S1). Connectivity with frontoparietal regions but not with limbic regions was negatively moderated by impulsivity. Specifically, healthy controls high in impulsivity, as measured by the UPPS Negative Urgency Scale, showed lower connectivity between vmPFC and lateral frontoparietal regions (inferior frontal gyrus and superior and inferior parietal lobules; Fig. 2b right; Table S2). Effects of impulsivity on connectivity were specific to these lateral cortical areas; subcortical and medial cortical regions showed persistent connectivity with vmPFC value signals in all subjects and were not affected by impulsivity. In addition, only impulsivity, but not indices of estimated intelligence quotient (IQ) or executive function, moderated vmPFC connectivity. Right and left striatum also showed strong connectivity during value updating, but this connectivity was not moderated by impulsivity (Fig. S2b).

We next investigated whether these normative effects of impulsivity on vmPFC–frontoparietal connectivity found in the nonpsychiatric controls were also present in the patient groups. Regions from the nonpsychiatric control group were used as an independent functional mask to test the relationship between vmPFC–frontoparietal connectivity and impulsivity in the patient groups. This relationship varied significantly by group, such that participants with a history of suicide attempts showed a significantly reduced relationship between impulsivity and vmPFC connectivity to frontoparietal regions compared to depressed controls (Fig. 2c; interaction effect of impulsivity by group from analysis of variance: *F*_2,73_ = 4.69, *p* = 0.01; *t*-statistic vs. depressed controls: *t*_73_ = −3.06, *p* < 0.005). Other individual characteristics, including estimated IQ and executive function, did not show this disrupted relationship with vmPFC–frontoparietal connectivity; this relationship was also not moderated by vmPFC value signals (all *F*s < 2.0 and all *p*s > 0.1). In right and left striatum, impulsivity-modulated regions in controls (defined using a lenient threshold of *t* < 2.0 due to non-significant whole-brain results) also did not show modulation of connectivity strength by group status (Fig. S2c; interaction effect of compulsivity by group: right striatum *F*_2,73_ = 0.628, *p* > 0.5; left striatum *F*_2,73_ = 0.906, *p* > 0.4).

### Brain–behavior relationships

**Fig. 3:**
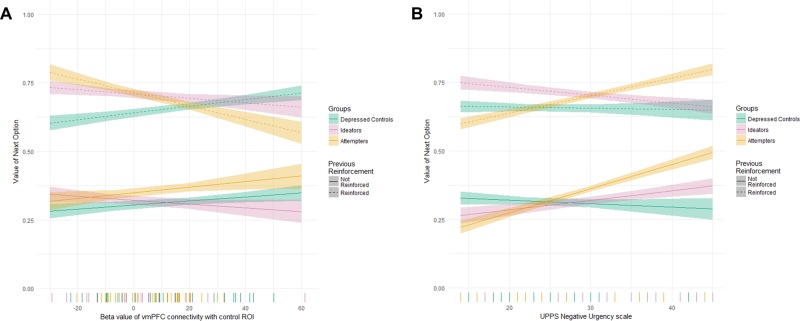
Effect of altered vmPFC–frontoparietal activity on behavioral performance. *X* axis is vmPFC–frontoparietal connectivity, *Y* axis is the value of the next choice (as calculated by the computational model), and line types indicate the presence of reinforcement on the current trial. In non-suicidal depressed participants, higher vmPFC–frontoparietal connectivity is associated with choosing better (higher valued) choices after both reinforced and non-reinforced trials, while participants with a history of suicide attempts show a breakdown in performance with high connectivity after reinforced trials.

To understand the behavioral relevance of vmPFC–frontoparietal connectivity on the quality of choices, we focused on the effect of previous trial reinforcement on expected value of the current choice and its interaction with vmPFC–frontoparietal connectivity and group. Allowing group status to modulate the relationship between the previous trial’s reinforcement and vmPFC–frontoparietal connectivity on the value of choices significantly improved model fit (*χ*^2^_6_ = 55.95, *p* < 0.001). Specifically, participants with a history of suicide attempts showed a disrupted relationship between vmPFC–frontoparietal connectivity and previous reinforcement on subsequent choice values, relative to other clinical groups (Fig. 3a; 3-way interaction of vmPFC–frontoparietal connectivity, group status, and previous reinforcement: *χ*^2^_2_ = 37.68, *p* < 0.001; *t*-statistic vs. depressed controls: *t* = 6.01, *p* < 0.001). This effect was robust to excluding participants with possible brain damage, current or lifetime substance use, or treatment with antipsychotic medication. This result indicates that, while participants in the depressed control and ideator groups showed a positive relationship between vmPFC–frontoparietal connectivity and choice value after both rewarded and non-rewarded trials, suicide attempters failed to benefit from recent rewards, particularly if they displayed high vmPFC–frontoparietal connectivity. Therefore, participants with a history of suicide attempts showed a reduced modulation of the effect of previous reinforcement on the value of choices by impulsivity-related neural connectivity with vmPFC signals.

## Discussion

We aimed to examine the relationship between impulsivity and neural indices of disrupted decision processes in older adults with suicidal behavior. We found that value signals in vmPFC, a central region in value-based decision-making, were reduced in suicide attempters vs. healthy controls with intermediate levels in patient comparison groups. Functional connectivity between vmPFC and frontoparietal cognitive control areas during value updating was negatively moderated by impulsivity among the comparison groups, but this relationship was absent among suicide attempters. Behaviorally, whereas stronger vmPFC–frontoparietal connectivity in comparison groups predicted higher-valued choices, in suicide attempters it predicted lower-valued choices following recent rewards. These results were specific to the vmPFC and not to the striatum. Together, these results point to an altered pattern of vmPFC–frontoparietal connectivity in impulsive people with suicidal behavior, distinct from that seen in non-suicidal impulsive individuals and associated with disrupted choice processes. This constellation may mark a neurobiologically distinct subtype of suicidal behavior.

Postmortem and imaging studies of suicide behavior implicate ventral prefrontal cortex [34, 35], although the literature remains sparse and inconsistent [36]. As in our previous study of attempted suicide [4], we found blunted value signals in vmPFC, potentially supporting a role of altered vmPFC value representation in suicidal behavior even in more complex decision-making scenarios. However, in this sample suicide attempters differed only from non-depressed controls but not from patient comparison groups. This pattern suggests that people who attempt have reduced value representation in vmPFC, but finding differences relative to patient groups may require larger sample sizes or accounting for yet-uncaptured moderators. This result is nevertheless informative given that group differences were detected in an unbiased, meta-analytically defined vmPFC ROI.

Reduced vmPFC–frontoparietal connectivity has previously been linked to self-report and behavioral measures of impulsivity [14–16], a pattern observed in our comparison groups. Importantly, in the non-suicidal groups, effects of impulsivity on vmPFC connectivity were regionally selective. Higher impulsivity was associated with weaker vmPFC connectivity with frontoparietal regions involved in cognitive control, while limbic and para-limbic connectivity (with striatum, hippocampus, posterior cingulate/precuneus) was spared. This regional specificity parallels the general rostral–caudal connectivity gradient in the vmPFC with greater limbic connectivity in subgenual cortex and more lateral prefrontal connectivity in perigenual cortex [37]. Supporting the notion that intact vmPFC–frontoparietal connectivity supports greater cognitive control over decision processes, it was associated with better (higher-valued) choices on the task. Whereas among the comparison groups vmPFC connectivity with frontoparietal cognitive control networks was moderated by trait impulsivity, this effect was abolished in suicide attempters. At a high level, our present findings echo those of our earlier study, which found that impulsivity moderated vmPFC representation of value [4]. Here the more cognitively demanding decision task revealed an effect of impulsivity on vmPFC connectivity rather than on value-dependent activation; self-reported impulsivity also did not differ between attempters and ideators in this sample. In summary, negative urgency-related impulsivity may involve reduced cognitive control over learning and choice processes, as expressed in diminished vmPFC–frontoparietal connectivity when updating value estimates during reinforcement learning.

In people who had attempted suicide, vmPFC–frontoparietal connectivity during value updating was not moderated by impulsivity nor did it facilitate a behavioral preference for higher-valued choices. Taken together, these observations suggest that vmPFC–frontoparietal connectivity in suicide attempters reflects abnormal encoding of reinforcement. Impulsivity along with suicidal behavior may be a neurobiologically distinct from other forms of impulsivity, characterized by functionally abnormal, rather than simply reduced, vmPFC–frontoparietal connectivity leading to an inability to make optimal decisions. This suggests that people who attempt suicide display intact connectivity, in contrast to non-suicidal people high in impulsivity. However, as shown by its impact on behavior, the information communicated by this intact connectivity is abnormal. Impulsivity in suicide attempters could also result from yet-to-be-defined neural abnormalities distinct from vmPFC–frontoparietal connectivity; abnormalities in basal ganglia or amygdala are possible candidates [38–43]. Our results add to previous findings of impaired valuation and choice processes, and moderation of these impairments by impulsivity in suicidal behavior [4], and go beyond earlier work in using a task that better isolates value signals and is more sensitive to cognitive control demands.

How might altered vmPFC value signals and neurobiologically distinct mood-dependent impulsivity contribute to suicidal behavior during real-life decision-making? Our studies in several independent samples of older suicide attempters suggest a multiple-hits account of the decision diathesis to suicide. We see independent and co-occurring deficits in learning from reinforcement and value-based choice. Our results suggest that impulsivity has distinct neural signatures in people who have attempted suicide and is related to the inability to place recent experiences into context when making decisions. This impairment, combined with the inability to correctly represent values, may [1] distort how accumulating stressors are appraised, leading one to experience manageable problems as catastrophic, and [2] inflate the attractiveness of escape in such a crisis.

Strengths of this study include a carefully characterized sample, multiple comparison groups including suicide ideators, the use of model-based neuroimaging, and its integration with behavioral analyses. Limitations include a cross-sectional design and inability to include older and more cognitively impaired participants due to the demands of the imaging protocol. Aside from sampling variability, reasons for divergence from our earlier study with a serial reversal task may include a more complex value-based decision-making task with constantly varying, highly uncertain contingencies and the lack of well-differentiated reciprocal updates or reversals. This design may have affected the strength or nature of value-related signals, potentially suggesting a trade-off between individual differences in value processing that are more pronounced in environments with stable and clearly differentiated option values and effects related to cognitive control and impulsivity that are more pronounced in environments with more volatile and less differentiated option values.

In summary, we found a distinct pattern of neural value representations in vmPFC and of vmPFC–frontoparietal connectivity in older adults who have attempted suicide. This evidence extends previous work of altered vmPFC value signaling in suicide by showing disrupted impulsivity-related modulation of value-related connectivity between the vmPFC and frontoparietal control regions in suicide. People who had attempted suicide displayed an abnormal pattern of vmPFC–frontoparietal connectivity, with reduced modulation by impulsivity and negative effects on learning.

## Funding and disclosure

This work was funded by the National Institutes of Health, Bethesda, MD, USA (R01MH100095 and R01MH048463 to AYD; K01MH097091 to MNH; R01MH085651 to KS; and T32MH019986 to VMB). The funding agency had no role in the design and conduct of the study; the collection, management, analysis, and interpretation of the data; preparation, review, or approval of the manuscript; or decision to submit the manuscript for publication. The authors do not have any conflicts of interest to disclose.

## Supplementary information

Supplemental material
