# Supplementary material for: Ventromedial prefrontal value signals and functional connectivity during decision-making in suicidal behavior and impulsivity
Source: Neuropsychopharmacology. 2020 Feb 8;45(6):1034–41. doi: 10.1038/s41386-020-0632-0 (PMC7162923; doi:10.1038/s41386-020-0632-0)
Supplement: Supplementary file 1 — Supplemental material [file 41386_2020_632_MOESM1_ESM.docx]

**Supplemental Information**

Table S1: vmPFC connectivity at time of feedback in non-psychiatric controls

Table S2: vmPFC connectivity and impulsivity (UPPS Negative Urgency) interaction in non-psychiatric controls

Figure S1: Task performance by group

Figure S2: Striatal PPI connectivity and moderation by impulsivity.

Supplementary text:

Participant inclusion/exclusion details

Measures used in sensitivity analyses.

Behavioral model fitting and performance

fMRI data collection, preprocessing, and first-level analysis

Supplementary References

Table S1. vmPFC connectivity at time of feedback in non-psychiatric controls

| **Cluster Number** | **Number of Voxels** | **Peak Coordinates (MNI space)** | | | **Peak t Value** | **Anatomical Label at Peak** |
| --- | --- | --- | --- | --- | --- | --- |
|  |  | **x** | **y** | **z** |  |  |
| 1 | 109,993 | -3 | -54 | 26 | 13.74 | Posterior cingulate cortex |

Table S2. vmPFC connectivity and impulsivity (UPPS Negative Urgency) interaction in non-psychiatric controls

| **Cluster Number** | **Number of Voxels** | **Peak Coordinates (MNI space)** | | | **Peak t Value** | **Anatomical Label at Peak** |
| --- | --- | --- | --- | --- | --- | --- |
|  |  | **x** | **y** | **z** |  |  |
| 1 | 542 | 31 | -65 | 51 | 3.79 | Right superior parietal lobule |
| 2 | 295 | -33 | -65 | 49 | 3.66 | Left superior parietal lobule |
| 3 | 275 | 45 | 11 | 22 | 3.62 | Right inferior frontal gyrus |

All results are reported using a cluster-forming threshold of p < .001 and thresholded at p < .05 FWE-corrected for false positives using nonparametric correction.


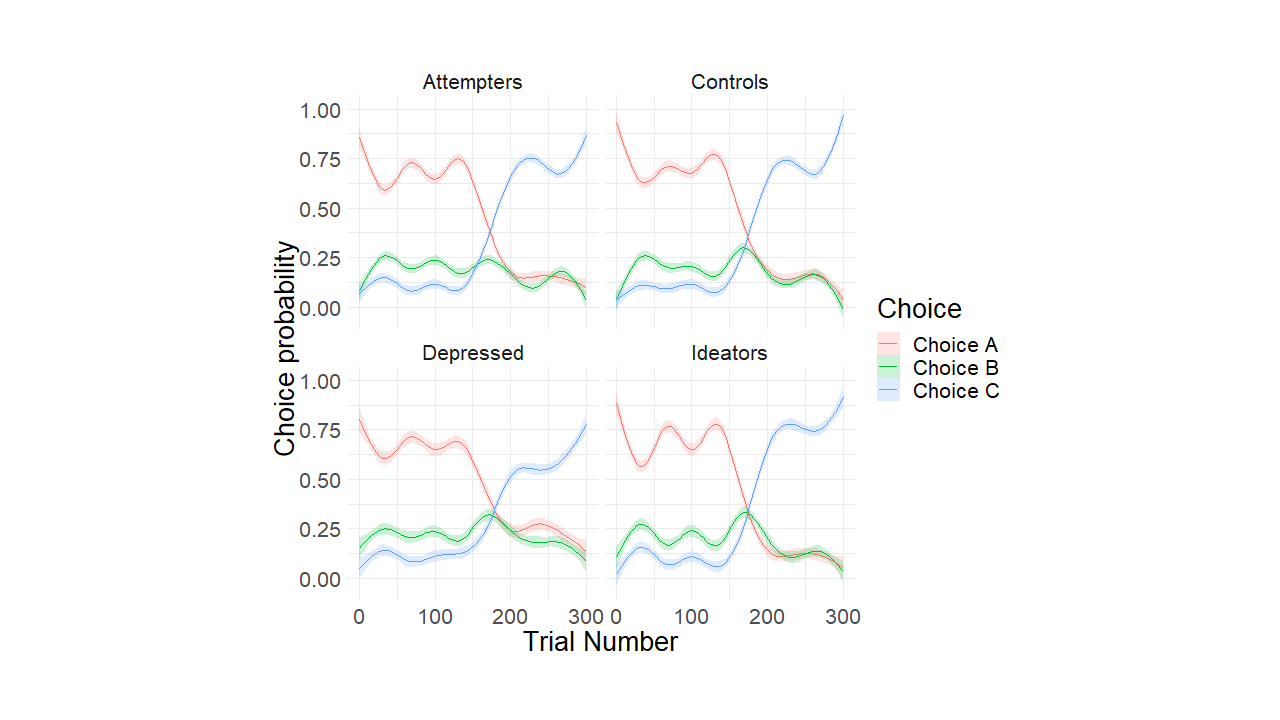


Figure S1: Task performance by group. This figure displays performance as in Figure 1C, broken down by group.


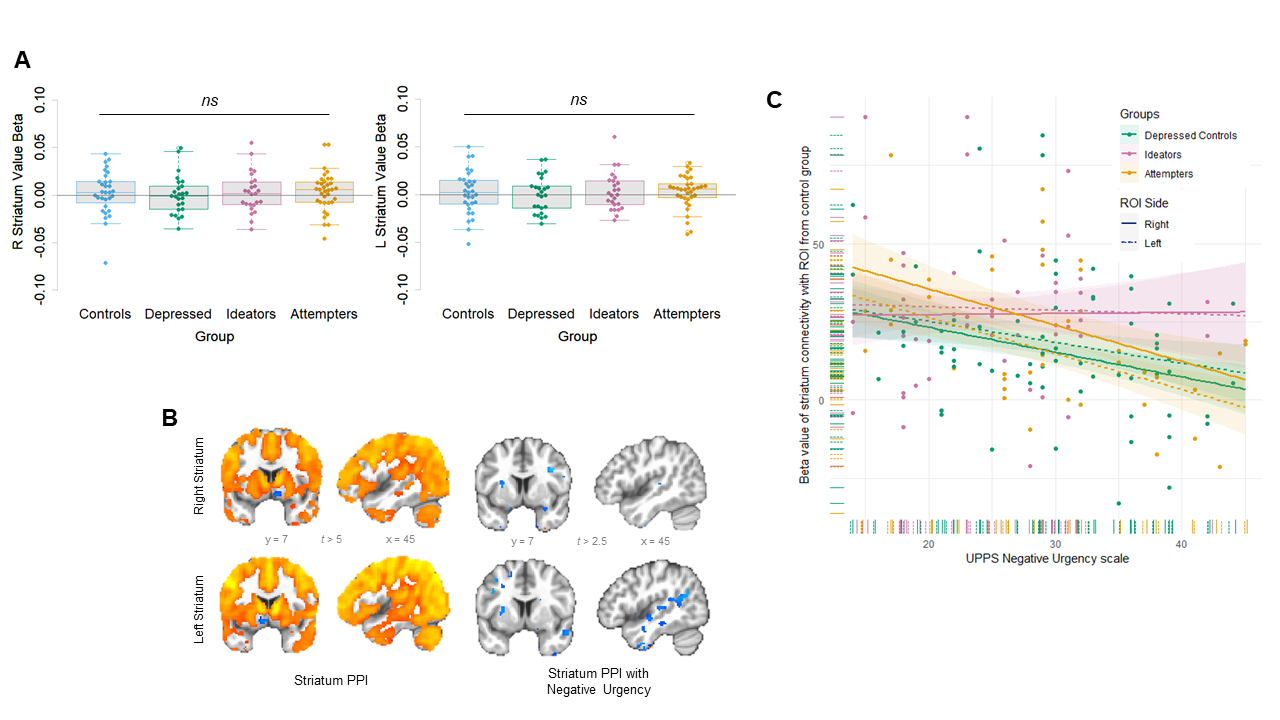


Figure S2: Striatal PPI connectivity and moderation by impulsivity. **A**: Right and left striatal value signals by group. Box plots are grouped by patient group and individual dots represent each participant in the group. Values shown are the average beta value for expected value in meta-analytically defined right and left striatum regions of interest. Striatal value signals did not differ by group in either right or left striatum (right striatum: all ts < 1.0, ps > 0.5; F_3,112_ = 0.117, p > .9; left striatum: all ts < 1.0, ps > 0.5; F- F_3,112_ = 0.123, p > 0.9). **B**: Striatal connectivity with value and moderated by impulsivity in nonpsychiatric controls. In controls, striatal signal at time of feedback was significantly correlated with activity in cortical and subcortical brain areas (left); but was not significantly moderated by impulsivity (right; results are shown at a lenient threshold but do not survive correction). **C:** Moderation of relationship between impulsivity-moderated connectivity with striatum by group. X axis represents impulsivity scores (UPPS Negative Urgency) and Y axis represents connectivity between striatum and region of interest showing altered connectivity with impulsivity in non-psychiatric controls (mask derived from regions shown in Figure 2B at a lenient threshold of t > 2.0). Dots indicate individual participants and lines indicate overall relationship per group; colors indicate patient groups and line types indicate right vs. left striatum seeds. Groups do not significantly differ in modulation of connectivity by impulsivity.

Supplementary text

Participant inclusion/exclusion details.

Exclusion criteria for all participants included: clinical dementia, as indicated by a diagnosis or score < 24 on the Mini-Mental State Examination [1], inability to perform a learning task, as indicated by a history of neurological disorder, delirium, or sensory disorder, and inability to undergo MR scanning, as indicated by the presence of ferrous metal, use of a pacemaker, or claustrophobia or other issues tolerating the scanning environment.

For participants with a history of suicide attempts, suicide attempt history was verified by a psychiatrist (AYD or KS) using all available information: the participant’s report, medical records, and information from the treatment team, family, and friends. If significant discrepancies existed between sources, the participant was excluded. There was no relationship between the length of time since the most recent attempt and current ideation (r = 0.068, p > 0.1). Twelve participants (seven non-psychiatric controls, three depressed controls, and two attempters) had also participated in our previous study on value-based decision-making in suicide [2]; excluding these participants did not meaningfully change results. No participants had documented brain damage from suicide attempts, but as medical record review could not rule out brain damage in 3/35 attempters, sensitivity analyses excluded these participants. Additional sensitivity analyses excluded participants taking antipsychotic medications or with current or lifetime substance abuse and assessed effects of lethality of attempts, early versus late onset of suicidal behavior (defined as first attempt before versus after age 50), length of time since the last reported attempt, number of attempts, and time between baseline and scanning assessments.

Measures used in sensitivity analyses.

The medical seriousness of attempts was assessed using the Beck Lethality Scale (BLS; [3]); for participants with multiple attempts, data for the highest-lethality attempt were used. Eight of 35 included participants with a history of suicide attempts had at least one high-lethality attempt (BLS score ≥ 4), with high-lethality attempts resulting in coma, need for resuscitation, unstable vital signs, penetrating wounds of abdomen or chest, third-degree burns, or major bleeding. Dementia severity was assessed with the Mattis Dementia Rating Scale [4] and cognitive control function with the Exit Interview [5].

Behavioral model fitting and performance.

Summaries of choice behavior showed that all participants were able to learn the task (mean/SD proportion correct by group [chance = 0.33]: attempters 0.538/0.111; ideators 0.558/0.096; depressed controls 0.498/0.118; nonpsychiatric controls 0.571/0.100; F_1,99_ = 0.093, p > 0.1, where a correct choice was defined as choosing the option with the highest probability of reward). Participants expected to receive their cumulative wins at the end of the experiment, but to avoid penalizing poorly performing participants, the final payoff was fixed at $15.

Behavioral model fitting followed methods described in [6] and are briefly summarized here. To create trial-wise estimates of learned value, a Q-learning model was estimated from participants’ choices and outcomes using an empirical Bayesian implementation of the Variation Bayesian Approach, using the VBA-MFX approach in the VBA toolbox in Matlab (<http://mbb-team.github.io/VBA-toolbox/wiki/VBA-MFX/>); [7]. Parameters for all participants were estimated in the same model with one group-level prior.

To determine the best fitting model to behavior, models with and without parameters for 1) separate learning rates for rewards and omissions (positive and negative learning rate), 2) decay of unchosen options, and 3) choice autocorrelation were tested and compared against a null model and a win-stay/lose-shift model. Bayesian model comparison tested the relative fit of each model, allowing for individual heterogeneity in model fits, and the Bayesian Omnibus Risk (BOR) of the probability of the best fitting model was calculated [8].

The relative evidence for different models can be characterized using random effects Bayesian model comparison (BMC), while accounting for the full statistical risk incurred (Bayesian omnibus risk, BOR) [9]. The best fitting model is described by the below equations and included parameters for positive and negative learning rates, decay of unchosen options, and choice stochasticity (estimated model frequency = 0.769, BOR < 10^-31^).

Q-learning model:

where eq. 1 is the standard delta learning rule for updating action values *Q* at time *t*; eq. 2 specifies different learning rates for rewards and reward omissions; eq. 3 enables the decay (forgetting) of the values of unchosen stimuli controlled by the decay parameter λ; and eq. 4 is the standard softmax choice rule with a temperature (stochasticity) parameter β.

Participants’ parameters were fit to this model and values of options at each trial were calculated based on these parameters and their history of choices and outcomes. These values were then used as regressors in fMRI analyses described in the main text.

fMRI Data Collection, Preprocessing, and First-Level Analysis

Participants were scanned on a 3T Siemens Trio MR scanner fitted with a 32-channel head coil. Three runs of simultaneous multislice-accelerated echoplanar images (40 slices, voxel size = 3.125 mm^3^, flip angle = 45°, TR = 750 ms, TE = 30 ms, FOV = 200 mm, matrix = 64 x 64, multiband acceleration factor of 5, anterior-to-posterior phase encoding direction) were collected at a 30° hyperangulation from the AC-PC line while participants performed the learning task. To aid in registration and to quantify local magnetic field inhomogeneity, a high-resolution anatomical MPRAGE T1 scan (voxel size = 1 mm^3^, flip angle = 9°, TR = 2.3 s, TE = 3.43 ms, FOV = 256 mm, matrix = 256 x 256) and a two-echo gradient echo fieldmap (TE1 = 4.92 ms, TE2 = 7.38 ms) were collected.

Preprocessing was conducted with a custom pipeline using FSL and AFNI and consisted of: slice timing correction, motion correction, co-registration to the structural image, intensity thresholding, normalization and nonlinear transformation to MNI space, smoothing (8 mm FWHM), high-pass filtering, and intensity normalization. To reduce distortion due to susceptibility artifacts, particularly near the vmPFC, we applied a fieldmap correction implemented in FSL FUGUE to the functional data. This was implemented as part of a one-step interpolation that transformed subjects’ data into MNI152 template space. More specifically, the alignment of subjects’ functional images to their anatomical scan was computed using the white matter segmentation of each image and a boundary-based registration algorithm [10], augmented by fieldmap unwarping coefficients estimated by FUGUE. Given the low contrast between gray and white matter in echoplanar scans with fast repetition times, we first aligned functional scans to a single-band fMRI reference image with better contrast. The reference image was acquired using the same scanning parameters, but without multiband acceleration. Finally, functional scans were warped into MNI152 template space (3mm resolution) in a single step using the concatenation of functional-reference, fieldmap unwarping, reference-structural, and structural-MNI152 transforms.

Participants with poor fMRI image quality, who were not able to complete the scan due to discomfort, or with data lost due to scanner malfunctions or data collection issues (n = 19) were excluded from analyses. For participants included in analyses, framewise displacement (FD) was calculated and volumes with FD greater than 0.9 mm were censored during analysis [11].

Voxelwise general linear models (GLMs) were estimated with AFNI with autocorrelation accounted for by an ARMA(1,1) model. Similar to previous work[2], subject-level analyses included regressors for expected value (calculated from the computational model described above) as a parametric modulator at the time of feedback as well as regressors for trial onset, button press, run number, and censored high-motion volumes. Using the maximum available value at the time of feedback allowed for assessment of value-related processes at the time value was updated and in a manner that was insensitive to noise in model fitting or in participants’ decisions.

Supplementary References

1. Folstein MF, Robins LN, Helzer JE. The Mini-Mental State Examination. Arch Gen Psychiatry. 1983;40:812.

2. Dombrovski AY, Szanto K, Clark L, Reynolds CF, Siegle GJ. Reward signals, attempted suicide, and impulsivity in late-life depression. JAMA Psychiatry. 2013;15213.

3. Beck AT, Beck R, Kovacs M. Classification of suicidal behaviors: I. Quantifying intent and medical lethality. Am J Psychiatry. 1975;132:285–287.

4. Mattis S. Dementia Rating Scale (DRS). Odessa, FL: Psychological Assessment Resources; 1988.

5. Royall DR, Mahurin RK, Gray KF. Bedside Assessment of Executive Cognitive Impairment: The Executive Interview. J Am Geriatr Soc. 1992;40:1221–1226.

6. Dombrovski AY, Hallquist MN, Brown VM, Wilson J, Szanto K. Value-based choice, contingency learning and suicidal behavior in mid-life and late-life depression. Biol Psychiatry. 2018. 2018. https://doi.org/10.1016/j.biopsych.2018.10.006.

7. Daunizeau J, Adam V, Rigoux L. VBA: A probabilistic treatment of nonlinear models for neurobiological and behavioural data. PLoS Comput Biol. 2014;10.

8. Rigoux L, Stephan KE, Friston KJ, Daunizeau J. Bayesian model selection for group studies - Revisited. Neuroimage. 2014;84:971–985.

9. Rigoux L, Stephan KE, Friston KJ, Daunizeau J, Penny WD, Moran RJ. Bayesian Model Selection for group studies. Neuroimage. 2009;46:1004–1017.

10. Greve DN, Fischl B. Accurate and robust brain image alignment using boundary-based registration. Neuroimage. 2009;48:63–72.

11. Siegel JS, Power JD, Dubis JW, Vogel AC, Church JA, Schlaggar BL, et al. Statistical improvements in functional magnetic resonance imaging analyses produced by censoring high-motion data points. Hum Brain Mapp. 2014;35:1981–1996.
